# Frequency of asymptomatic carriers of SARS-CoV-2 among children and adults after school reopening

**DOI:** 10.1186/s13052-021-01016-5

**Published:** 2021-03-12

**Authors:** Gregorio P. Milani, Paola Marchisio, Alessia Rocchi, Giuseppe Bertolozzi, Ludovico Furlan, Adriano La Vecchia, Carlo Agostoni, Giorgio Costantino

**Affiliations:** 1 Pediatric Unit, Fondazione IRCCS Ca’ Granda Ospedale Maggiore Policlinico, Milan, Italy; 2 Department of Clinical Science and Community Health, University of Milan, Milan, Italy; 3 Department of Pathophysiology and Transplantation, University of Milan, Milan, Italy; 4 Dipartimento di Emergenza e Urgenza, Fondazione IRCCS Ca’ Granda Ospedale Maggiore Policlinico, Milan, Italy

## Abstract

**Background:**

Children often develop an asymptomatic form of Severe Acute Respiratory Syndrome Coronavirus 2 (SARS-CoV-2), but it is debated if children are at higher risk than adults to be asymptomatic carriers of SARS-CoV-2, especially during the school reopening. The main aim of this study was to investigate the frequency of SARS-CoV-2 asymptomatic carriers in children and adults during the reopening of the schools in Milan, Italy.

**Methods:**

We conducted a cross-sectional study at the pediatric and adult Emergency Department (ED) of the Ca' Granda Ospedale Maggiore Policlinico (Milan) between October 1 and 31, 2020, i.e. 3 weeks after the reopening of schools. Patients admitted to the ED short stay observation and without any sign or symptom consistent with a SARS-CoV-2 were eligible. These patients underwent a nasopharyngeal swab specimen for the detection of SARS-CoV-2. The odds ratio and its 95% confidence interval (CI) was calculated to assess the risk of asymptomatically carrying the SARS-CoV-2 infection in children and adults.

**Results:**

A total of 69 (27 females, median age 8.7 years) children and 251 (107 females, median age 71 years) adults were enrolled. Pediatric and adult subjects tested positive for SARS-CoV-2 with a similar frequency (1/69 [1.4%] vs 4/251 [1.6%]). Children had an odds ratio to be a carrier of 0.91 (CI 0.02– 9.38) compared to adults.

**Conclusions:**

The frequency of asymptomatic SARS-CoV-2 carriers was similar among children and adults. Considering the emerging diffusion of new SARS-CoV-2 variants, the asymptomatic spread of SARS-CoV-2 infection among children and adults should be monitored.

## Background

School closure has been adopted by many countries to limit the spread of Severe Acute Respiratory Syndrome Coronavirus 2 (SARS-CoV-2) [1]. One rationale for school closures is that children may asymptomatically spread the virus among their peers and school staff [2]. Whether such a measure is effective for this purpose is still debated [3]. The few available data from other parts of Italy have suggested a non-negligible risk of transmission of SARS-CoV-2 in the schools [4], while other studies conducted in other European Countries such as Germany [5] and Ireland [6] have reported a very low risk of secondary infection from children attending schools. In a previous study conducted during the school closure for the first SARS-CoV-2 outbreak in Italy, we observed a lower frequency of asymptomatic carriers among children as compared to adults (1.2% vs 9.2%, respectively) in hospital settings [7]. However, no comparable data are available after the school reopening. The purpose of this study was to investigate the frequency of SARS-CoV-2 asymptomatic carriers in children and adults during the reopening of the schools in Milan (Italy). Moreover, we investigated if there was a different frequency of asymptomatic carriers among children during the school re-opening and the school closure period.

## Methods

This study was conducted at the pediatric and adult Emergency Department (ED) of the Ca' Granda Ospedale Maggiore Policlinico (Milan, Lombardy) between October 1 and 31, 2020, i.e. 3 weeks after the reopening of schools. Since Lombardy was severly affected by the pandemic, accounting for about 30% of all nationwide infections, well-defined procedures were implemented to contain in-hospital spread of the SARS-CoV-2 infection. In particular, two clinical pathways were established in the Ca' Granda Ospedale Maggiore Policlinico for non-suspected and suspected cases of SARS-CoV-2 infection, to avoid any potential contact (and infection spread) between these two groups of patients [8]. A nasopharyngeal swab specimen was collected for the detection of SARS-CoV-2 in all patients admitted in the short stay observation of the ED, irrespective of their clinical presentation [7]. The swab was sampled by a trained nurse according to a standardized procedure [9].

For the purposes of this study, all children and adults admitted to the ED for at least 12 h were eligible. Patients with symptoms or signs consistent with SARS-CoV-2 infection were not included. Nasopharyngeal swab specimens were sent within 30 min to the Central Laboratory of the Hospital and immediately analyzed by multiplex real-time reverse transcription polymerase chain reaction targeting three (E, RdRP and N) SARS-CoV-2 genes [10].

The following data were prospectively collected and recorded in a predefined database: age, sex and reason for hospitalization in the short stay observation. Moreover, the development of symptoms possibly consistent with the SARS-CoV-2 infection in asymptomatic carriers was monitored in the following 2 days (clinically or by a telephone call).

The two-tailed Fisher’s test was applied to compare the proportions between the two populations (children vs adults). The odds ratio and its 95% confidence interval (CI) was calculated to assess the risk of asymptomatically carrying the SARS-CoV-2 infection. Moreover, we also compared the frequency of asymptomatic children observed during the school closure [7] with that obtained after reopening of the schools. Significance was defined for a *p* < 0.05. The study was approved by the Ethics Board of the Hospital.

## Results

During the study period, there were 1047 visits at the pediatric ED. A total of 69 children (27 females, median age  8.3 years) were eligible for the study. In adult ED, there were 2511 visits and 251 (107 females, median age 71 years) fulfilled the criteria to participate. Table 1 shows the reasons for the hospitalization of the included patients. Children and adults tested positive for SARS-CoV-2 with a similar frequency (1/69 [1.4%] vs 4/251 [1.6%] respectively, *p* = 1.0). Children had an odds ratio to be a carrier of 0.91 (CI 0.02–9.38) compared to adults. None of the included patients developed a symptomatic infection in the 2 days following the test. No difference in the frequency of asymptomatic carriers among children was observed during the school reopening as compared to that observed during the school closure (1/69 [1.4%] vs 1/83 [1.2%], respectively, *p* = 1.0). The odds ratio to be carriers of SARS-CoV-2 among children was not significantly different during school re-opening (1.20, CI 0.02–95.73) compared to the period of school closure.

**Table 1:** Characteristics of children and adults (total = 320) included in the study during the school re-opening. Data are presented as median [interquartile range] or as frequency (percentage)

	Children	Adults
N	69	251
Male	42 (61)	144 (57)
Age (years)	8.3 [1.0–16]	71 [56–81]
Positive for SARS-CoV-2	1 (1.4)	4 (1.6)
Reason for hospital admission		
*Surgical intervention*	11 (16)	43 (17)
*Neurologic disease*	14 (20)	56 (22)
*Trauma*	9 (13)	24 (9.6)
*Cardiac disease*	3 (4.3)	19 (7.6)
*Psychiatric disorder*	4 (4.3)	9 (3.6)
*Intoxication*	5 (5.8)	3 (1.2)
*Other conditions*	23 (33)	97 (39)

## Discussion

This study points out that the frequency of asymptomatic carriers of SARS-CoV-2 among children and adults is similar after the reopening of the schools in Milan, one of the epicenters of the second wave of the pandemics in Italy. Moreover, a similar frequency of asymptomatic carriers was observed between the periods of school re-opening and school closure.

Increasing data suggest that adults, who asymptomatically carry the SARS-CoV-2 in the upper airways, might spread the infection [11]. On the other hand, it has been speculated that children play a key role in the SARS-CoV-2 transmission because, unlike adults, they do not frequently develop a notable disease [12]. The results of this survey do not support this assumption.

This study did not find any significant difference in the frequency of pediatric asymptomatic carriers during the school re-opening and the school closure.

Considering that children are likely to be less contagious than adults [13, 14], the burden of the school closure and confinement on their life [3, 15] and that data about a possible spike in SARS-CoV-2 cases during school reopening are contrasting [16], the cost-effectiveness of this measure should be carefully pondered by policy makers.

This study has limitations: the sample size and the frequency of asymptomatic carriers was rather little in both in children and in adults. Moreover, the data were obtained in a single center. We did not collect and compare data about symptomatic children and adults. Finally, the median age of adults was higher than the that of the general adult population. Although this finding is expected for subjects enrolled in the context of an emergency department, this might limit the generalizability of our results. This study has also strengths: most previous studies have assessed the frequency of asymptomatic carriers of SARS-CV-2 infection among children and adults in well-defined geographical areas [17] or in subjects sharing the same environment, such as the childcare facilities [5]. However, these studies were focused on the evaluation of possible infection outbreaks in the context of a defined community. This study provides an estimate of the asymptomatic carriers among children and adults in unselected random sample of children and adults. In addition, we addressed efforts to identify any possible pre-symptomatic infected subject by means of a follow-up.

## Conclusions

The frequency of asymptomatic SARS-CoV-2 carriers among children and adults was similar after the reopening of the schools in Milan. Considering the increasing number of new SARS-CoV-2 variants detection [18], the asymptomatic spread of SARS-CoV-2 infection among children and adults should be carefully monitored.

## Data Availability

Data are available at the corresponding author upon reasonable request.

## Abbreviations

SARS-CoV-2: Severe Acute Respiratory Syndrome Coronavirus 2
ED: Emergency Department
